# Reduced Carboxylate Graphene Oxide based Field Effect Transistor as Pb^2+^ Aptamer Sensor

**DOI:** 10.3390/mi10060388

**Published:** 2019-06-11

**Authors:** Fang Li, Zhongrong Wang, Yunfang Jia

**Affiliations:** College of Electronic Information and Optical Engineering, Nankai University, Tianjin 300071, China; lifang_6952@163.com (F.L.); 1120160100@mail.nankai.edu.cn (Z.W.)

**Keywords:** field effect transistor, reduced carboxylate graphene oxide, aptamer, sensor, screen print

## Abstract

Aptamer functionalized graphene field effect transistor (apta-GFET) is a versatile bio-sensing platform. However, the chemical inertness of graphene is still an obstacle for its large-scale applications and commercialization. In this work, reduced carboxyl-graphene oxide (rGO-COOH) is studied as a self-activated channel material in the screen-printed apta-GFETs for the first time. Examinations are carefully executed using lead-specific-aptamer as a proof-of-concept to demonstrate its functions in accommodating aptamer bio-probes and promoting the sensing reaction. The graphene-state, few-layer nano-structure, plenty of oxygen-containing groups and enhanced LSA immobilization of the rGO-COOH channel film are evidenced by X-ray photoelectron spectroscopy, Raman spectrum, UV-visible absorbance, atomic force microscopy and scanning electron microscope. Based on these characterizations, as well as a site-binding model based on solution-gated field effect transistor (SgFET) working principle, theoretical deductions for rGO-COOH enhanced apta-GFETs’ response are provided. Furthermore, detections for disturbing ions and real samples demonstrate the rGO-COOH channeled apta-GFET has a good specificity, a limit-of-detection of 0.001 ppb, and is in agreement with the conventional inductively coupled plasma mass spectrometry method. In conclusion, the careful examinations demonstrate rGO-COOH is a promising candidate as a self-activated channel material because of its merits of being independent of linking reagents, free from polymer residue and compatible with rapidly developed print-electronic technology.

## 1. Introduction

Aptamer functionalized graphene field effect transistor (apta-GFET) is an integrated sensing platform which can accommodate versatile sensing actions [1]; it fuses not only FET’s artful working principle [2,3] and graphene’s remarkable features [4], but also aptamer’s high sensitivity [5,6]. In contrast with other advanced channel materials in FETs, like Si nanowire [3], graphene oxide nano-ribbon [7], carbon nano-fiber [8], carbon nanotube (CNT) [9], the merits of graphene have still attracted considerable attention since its first report [10] because the graphene film can serve as both a highly conductive channel and a biocompatible substrate for grafting aptamer [11,12,13,14,15,16]. The efforts in these works are mainly focused on three aspects, which are the methods for graphene preparation, aptamer grafting and device fabrication. The graphene materials prepared by mechanical exfoliation [10,11] and chemical vapor deposition (CVD) [12,13,14,15] are widely used because of their perfect 2D-honeycomb carbon crystal structure, electronic field effect feature and bipolar character; however, their drawbacks of chemical inertness and polymer residues induced by transferring process cause difficulties in their applications. Recently, reduced graphene oxide (rGO) based on modified Hummers’ method has been exploited to fabricate an apta-FET biosensor with the purpose of developing a total liquid-chemical fabricating methodology [16]. It is demonstrated that rGO can be an acceptable solution to make GFET free from conventional transferring procedure. However, as to the method of functionalizing GFET with aptamer, to the best of our knowledge, all the studies have to introduce an extra linking process either on the graphene channel [10,12,13,15] or on aptamer molecules [11,14], based on hetero-bifunctional reagents like 1-pyrenebutanoic acid succinimidyl ester [10,15], glutaraldehyde conjugated 1,5-diaminonaphthalene [12,13], pyrene N-hydroxy succinimide [11] and pyrene phosphoramidite [14]. Even in the latest rGO based apta-FET, carboxylated CNT was still necessary to activate the rGO channel, so that the amino modified aptamer (anti-CA125) could be grafted on it [16].

Herein, an idea to develop a kind of self-activated GFET is proposed, for the purpose of tethering the aptamer molecules on the graphene channel forthright, without any extra linking processes. This self-activation idea originated from our previous work regarding carboxylated graphene oxide (GO-COOH), which has been demonstrated to be helpful in increasing the amount of immobilized anti-EpCAM and capturing more tumor cells [17]. We thought, if it is reduced, the reduced GO-COOH (rGO-COOH) would be a promising channel material. It has been demonstrated that even though modified Hummers method based rGO is not perfect, the recovered 2D-network can still serve as a channel material in the pH sensing GFET [18]. Still, we were still confronted with uncertainties as to whether the GO-COOH can be reduced to graphene-state and whether the carboxyl groups can be maintained after suffering reduction.

Furthermore, we would like to utilize the rapidly developed screen-print electronic technology to fabricate GFETs because of its inherent superiorities in commercial availability, suitability for large-scale use and low cost. Though it has been applied in many bio-assays [19,20,21], there are still some issues which needed to be addressed in this work. The method for depositing graphene derivatives (including rGO-COOH, its counterparts rGO and purchased graphene paste (pGp)) should be carefully chosen, and the order of process for forming the graphene channel should be designed as late as possible to avoid the possible negative influence caused by the following processes. The examinations including X-ray photoelectron spectroscopy (XPS), UV-visible (UV-vis) absorbance, atomic force microscopy (AFM) and scanning electron microscope (SEM), were carefully organized and carried out to demonstrate the of-interest rGO-COOH film is in few-layered graphene state and full of functional oxygen-containing groups.

Last but not least, the feasibility of the proposed rGO-COOH based GFETs as an aptamer sensor was evaluated using the lead specific aptamer (LSA, a kind of Pb^2+^ sensitive DNAzyme) as a proof-of-concept, because the imperceptible chronic intake of Pb^2+^ in water and foodstuff [22] can cause lots of toxicological effects on human health [23,24,25]. Pb^2+^ accumulation in basal ganglia can cause dysfunctions in the central and peripheral nervous system [26], Pb^2+^ induced defects in heme biosynthesis can invoke anemia [27] and porphyria [28], and clinical lead colic [29] is closely related with Pb^2+^ motivated oxidative stress in blood, liver and kidneys [30]. In this aspect, to prevent lead poisoning, Pb^2+^ detection techniques are necessary, such as conventional inductively coupled plasma mass spectrometry (ICP-MS), nano-material assisted colorimetric detection [31,32], UV-vis absorption [33] and stripping analysis [34,35]. LSA sensing strategy which is believed to be the Pb^2+^ caused cleavage effect at the position of ribonucleotide adenosine (rA) [5,6], has also been demonstrated to be available to functionalize field effect type sensors [11,36]. The parallel ICP-MS examinations, interference experiments for other metal ions (Cu^2+^, Mn^2+^, Mg^2+^, Hg^2+^, etc.), and the real applications for Pb^2+^ determinations in drinking water were executed step-by-step to evaluate the performances of the proposed rGO-COOH based apta-GFET as an aptamer sensor. The success of its application in Pb^2+^ could be extended to not only other heavy metal ion aptamers [5], but also tumor cell targeted aptamer probes (like AS1411, TSL11a, A10, etc.) [37].

## 2. Materials and Methods

### 2.1. Materials

**Figure 1:**
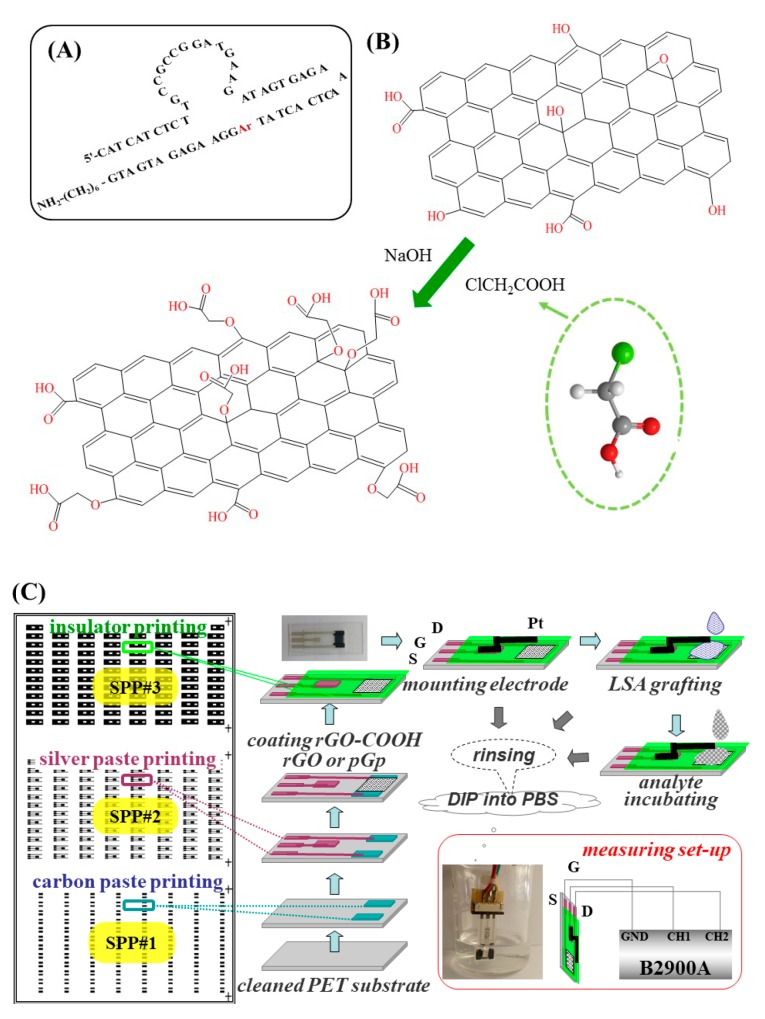
Protocol for the studies of reduced carboxyl-graphene oxide (rGO-COOH) based self-activated graphene field effect transistors (GFETs). (**A**) The nucleotides’ sequence and the molecular configuration of the lead specific aptamer used in this work. (**B**) The sketched principle for preparing GO-COOH. (**C**) A schematic representation for fabricating screen printed GFETs, the measuring set-up and the experimental procedure.

LSA is synthesized by Shanghai Sangon Inc. (China), its sequence is given in Figure 1A. In which, “rA” is the cleavage point, it is linked between the substrate chain GGAAGAGATGATG-(CH_2_)_6_-NH_2_-3’ and Pb^2+^ DNAzyme (5’-CATCATCTCTTGCCGCCG GATGAAGATAGTGAGAAACTCACTAT). When Pb^2+^ is absent from the analyte, the substrate chain and the DNAzyme string can be hybridized by their complementary parts to form a bulged intramolecular hairpin structure; on the contrary, in the presence of Pb^2+^, the hybridized string will be separated because of the Pb^2+^-mediated cleavage at rA site [6]. Meanwhile, the amino group (-NH_2_) at terminal 3’ is designed as the anchoring point, the carbon chain (-(CH_2_)_6_) close to -NH_2_ is used for increasing the flexibility of the LSA molecules and attenuating the possible hindering effect. In result, the substrate chain in LSA will be left on GFET after suffering Pb^2+^ cleavage.

The main chemicals for rGO-COOH and rGO, as well as fabricating devices, crystalline flake graphite powder (99%), NaOH (≥96%), KMnO_4_ (99.5%), H_2_SO_4_ (98.0%), H_2_O_2_ (30.0%), HCl (30.0%), hydrazine hydrate, liquid ammonia, hypochlorous acid (ClCH_2_COOH, 98.0%), of analytically pure grade, were purchased from Tianjin Chemical Reagent wholesale company (Tianjin, China). Polyethylene-terephthalate (PET) substrate (0.8 mm) was purchased from Guangdong Kuangye Plastic Material Co. Ltd. (Guangdong, China); 3-aminopropyltriethoxysilane (APTES) was from Sigma-Aldrich (St. Louis, MO, USA); the conductive silver paste, carbon paste and insulating paste were from Shenzhen Haori Electronic Material Co. Ltd. (Shenzhen, China); pGp was from Suzhou Hengqiu Graphene Technology Co. Ltd. (Suzhou, China). Piranha solution was prepared by 30 mL H_2_SO_4_ (98.0%) and 10 mL H_2_O_2_ (30.0%). De-ionized water (DIW) was used for all the chemical solutions’ preparation.

The main reagents prepared in the experiments were as follows: (1) The phosphate buffer saline (PBS) was Na_2_HPO_4_ and NaH_2_PO_4_ in DIW with the concentration of 100 mmol/L and pH was adjusted by HCl at about 7.4. (2) Tris-buffer solution was about 50 mmol/L and pH was adjusted at about 7.4. (3) APTES solution was in DIW with the volume ratio (*v*/*v*) of 1:10, pH 7.4. (4) LSA solutions were in PBS with the concentrations of 1 µmol/L, 500 nmol/L, 100 nmol/L, 50 nmol/L, 10 nmol/L, 5 nmol/L and 1 nmol/L, named as LSA_#i (i = 1, 2, 3, 4, 5, 6, 7). (5) The standard solutions of Pb^2+^ (Pb_std_) were prepared by diluting their standard samples in Tris-buffer solution with concentrations of 0.001, 0.01, 0.1, 1, 3, 5, 7, 10 ppb, named as Pb_std__#i (i = 1, 2, 3, 4, 5, 6, 7, 8). (6) The disturbing examinations were executed on the tris-buffer solutions of non-target metal ions (Cu^2+^, Mn^2+^, Mg^2+^, Al^3+^, Cr^2+^, Co^2+^, Hg^2+^, Ag^+^) (10 ppb), in contrast, the target ions concentration was 1 ppb. (7) Three kinds of drinking water, tap-water (TP), in-market purified water (PW) and mineral water (MW) were tested for Pb^2+^ determinations.

### 2.2. Methods and Principle for rGO-COOH Preparation

The preparation of rGO-COOH and rGO was based on the modified Hummers method [38]. The ground graphite powder suffered a series of harsh oxidations induced by H_2_SO_4_ (98%), KMnO_4_ and H_2_O_2_ (30%), which can damage the perfect sp^2^ conjugated 2D-carbon network and intersect oxygen into the tightly stacked layers; by which graphite oxide was produced. Starting from this byproduct, our concerned rGO-COOH and its counterpart rGO were prepared: (1) GO solution was obtained by extracting supernatant of the centrifugalized graphite oxide suspension (1 mg/mL); (2) GO-COOH was obtained by adding NaOH and ClCH_2_COOH into GO solution, centrifuging it at 12,000 rpm for 1 h, and extracting supernatant. This step converted OH groups and epoxy groups to -COOH groups, as depicted in Figure 1B. (3) The reductions of GO and GO-COOH were realized by the use of the hydrazine-hydrate and liquid ammonia reducing system [39]. It has been reported that by this method not only can the conjugated carbon structure can be recovered, but also the stable aqueous colloid can be obtained; good dispersion in water solution is attributed to the electrostatic repulsion (the negatively charged carboxyl groups on the edges of rGO nano-sheets). In this work, we used it to reduce the graphene precursor’s oxidation level, and maintain the carboxylated group as much as possible.

### 2.3. Methods of Device Fabrication

According to the printing electronic technique for FET devices [40], the procedures for the screen printed GFETs, named as SP-X-FETs (X = rGO-COOH, rGO and pGp), are sketched in Figure 1C and outlined here: (1) carbon paste was printed on the cleaned PET substrate, patterned by the first screen-print plate (#1) to form S and D, then dried in vacuum oven (100 °C) for 30 min; (2) Ag paste was printed by the #2 plate and dried (100 °C, 30 min) to form the conducting wires and electronic pads; (3) rGO-COOH and its counterparts (rGO and pGp) were coated by the method of drop-coating (for rGO-COOH and rGO) or knife-coating (for pGp because of its viscosity), respectively; then dried at 80 °C in vacuum oven for 1 h; (4) the insulating paste was printed by the #3 plate and dried at 70 °C in vacuum oven for 30 min. Once the fabrication of SP-X-FETs (X = rGO-COOH, rGO and pGp) was accomplished, one of them was photographed and shown in Figure 1C. After the Pt electrode was mounted, a series of examinations were executed. SP-X-FETs were functionalized by incubation with LSA solutions (40 μL) under optimized conditions, in the thermostatic oscillator (60 rpm, 37 °C) for 4 h. After rinsing off the un-anchored LSA molecules, these functionalized devices, named as apta-X-FET (X = rGO-COOH, rGO and pGp), were ready to be tested.

### 2.4. Apparatus

XPS examinations were executed by Axis Ultra DLD (Kratos Analytical Ltd., UK) to achieve wide and core XPS spectra of rGO-COOH, rGO and pGp films, for the purpose of identifying the surface components and their changes after being functionalized by LSA. Raman spectra were detected by micro-confocal Raman spectrometer RTS-HiR-AM (Titian Electro-Optics Co., LTD, Beijing, China), excited by laser with a wavelength of 532 nm. UV754N (Shanghai Precision Science Instrument Co., Ltd., Shanghai, China) was used for measuring the UV-vis absorbance of the synthesized graphene solutions of rGO-COOH and rGO. SEM S-3500N (Hitachi, Japan) and Dimension Icon (Bruck, USA) were used for taking the micro-morphological images. AFM experiments were conducted under the tapping mode. According to the reference [41], in this mode the thickness of one single graphene layer is about 0.95 nm. Digital source meter (DSM) B2900A (Agilent Technology Co., Ltd., Santa Clara, CA, USA) was utilized to perform electronic measurements of GFETs. ICP-MS examinations for Pb^2+^ determination were executed by Elan DRC-e (PerkinElmer, Waltham, MA, USA).

### 2.5. Electronic Set-Up and Measurements for SP-X-FET

The electronic examinations were executed by a typical three-electrode GFET testing system, as sketched in Figure 1C. The Pt electrode floated on and close to the surface of the graphene channel, it was the gate electrode (G). The other two electrodes S and D were connected directly to DSM. The routing of wires between three electrodes is diagrammed in Figure 1C, in which electrodes of S, D and G are connected to the DSM’s interfaces (GND, CH1 and CH2), respectively. The voltages between G and S, D and S are named as V_GS_ and V_DS_, respectively. The bias voltages of V_GS_ and V_DS_ were optimized and currents between S and D at these bias are named as I_DS,BLANK_. For the LSA modified devices, a cyclic experiment was designed and schemed in Figure S1A, that is: the device were incubated with LSA solutions in different concentrations (LSA_#i); after being incubated with each of LSA_#i, I_DS_ was measured and named as I_DS_LSA#i_; the change rate of I_DS_ was calculated by (I_DS_LSA#i_ − I_DS,BLANK_)/I_DS,BLANK_ and plotted in the coordinate of ΔI_DS_/I_DS_BLANK_ versus concentration of LSA (LSA_Conc.).

### 2.6. Detection of Pb^2+^ Standard Samples

The experiments were executed to evaluate the sensitivity, selectivity and reliability of the proposed self-activated SP-apta-FET. For each of apta-X-FETs (X = rGO-COOH, rGO and pGp), the operating steps were identical and repeated five times. The main procedures are outlined as follows: (1) It was dipped into PBS, as shown in Figure 1C the output currents were measured and named as I_DS,m0_. (2) After rinsing with DIW and dried naturally, it suffered from a cyclic detection of Pb^2+^ standard samples as sketched in Figure S1B, from Pb_std__#1 to Pb_std__#8. The operations for each of them were the same, which were dropping 30 μL Pb_std__#i on the channel surface, incubation in the thermostatic oscillator (60 rpm, 35 °C for 30 min), and then rinsing with DIW and measuring its I_DS,mi_ in the similar PBS environment. (3) The measured data were plotted as the curves of ΔI_DS_/I_DS,m0_ versus concentration of Pb_std__#i (Pb_Conc.), in which ΔI_DS_ = I_DS,mi_ − I_DS,m0_. Meanwhile, the ICP-MS examinations were executed on the similar Pb^2+^ standard samples. The electronic detection results and ICP data were plotted in a double y-axis coordinate.

### 2.7. Real Sample Examinations

The application of the proposed rGO-COOH based apta-GFETs was further evaluated by the real sample tests, using three kinds of drinking water as examples, which are the marketed pure water (PW), mineral water (MW), as well as tap water (TW) from municipal supply. The standard addition method (SAM), which is a classical analytical method and well-accepted to be able to eliminate matrix effect, was used in this experiment to determine Pb^2+^ concentrations. Details in this experiment are outlined as follows: (1) each of water samples was divided into six aliquots (10 mL). (2) Then, 0, 20, 40, 80, 100 μL standard Pb^2+^ solution (1000 ppb) was added into each of the aliquots. The added Pb^2+^ concentration (C_added-Pb_) should be 0, 2, 4, 6, 8, 10 ppb, respectively. (3) The apta-rGO-COOH-FETs were incubated with each of them, their output current deviations (ΔI_DS_) were measured and plotted in the coordinate plane of ΔI_DS_ versus C_added-Pb_. (4) Pb^2+^ concentrations in three samples were determined according to SAM, that is: the linear fitted lines were firstly achieved by the use of OriginPro 8.1, then their intersecting points at X-axis were calculated, the values of which were the concentrations of Pb^2+^ in each sample. (5) The conventional ICP-MS method was executed again on these real samples to make an assessment.

## 3. Results and Discussion

### 3.1. Identification and Morphology

**Figure 2:**
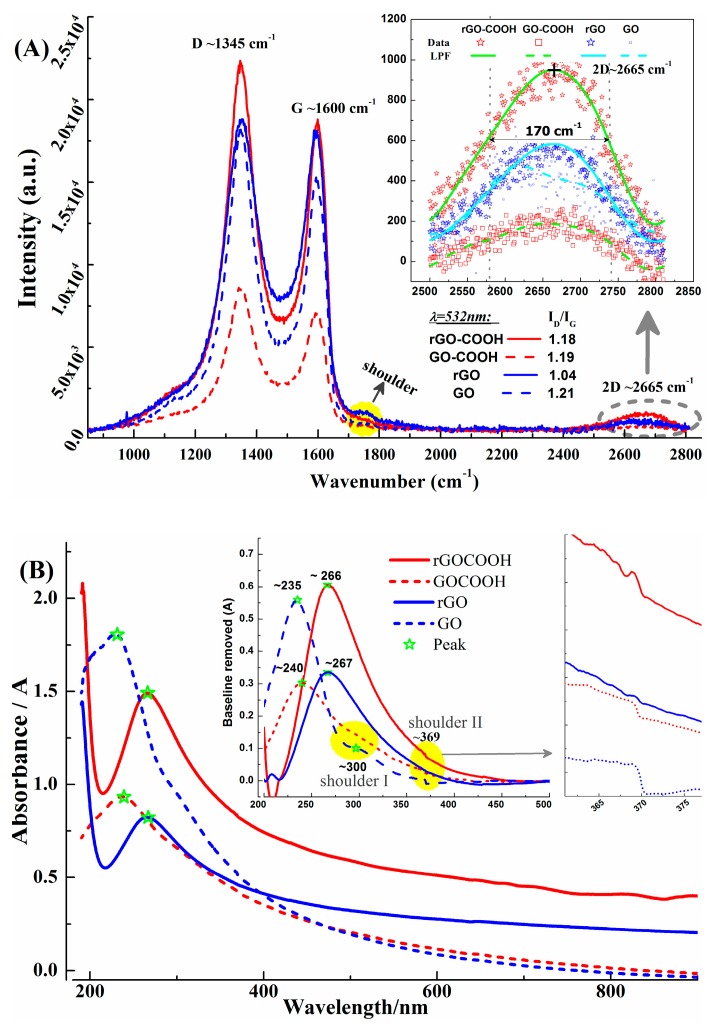

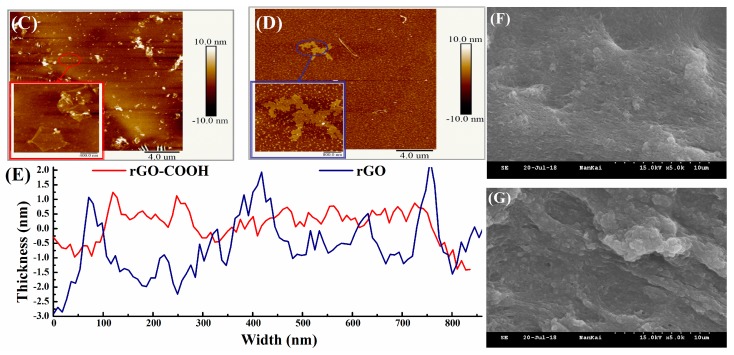
Identification of rGO-COOH. (**A**) Raman spectra of rGO-COOH in comparison with GO-COOH, rGO and GO (excited at λ = 532 nm). (**B**) UV-vis absorbance spectra. (**C**,**D**) AFM photos of rGO-COOH and rGO films at glass slides. (**E**) AFM analysis for film thickness. (**F**,**G**) SEM images of rGO-COOH and rGO films, respectively.

Raman spectrum is a fingerprint to recognize graphene [42], probe the defects in graphene [43] or GO [38], determine the removed functional groups from GO [44], as well as evaluate the efficiency of the reducing method [45]. Typical graphene Raman spectra can be recognized in Figure 2A, which are the peaks at ~2665 cm^−1^ falling into the region of the 2D band (2550–2900 cm^−1^) [43], the peaks at 1345 cm^−1^ belong to the D band [44], and the peaks at ~1600 cm^−1^ correspond to G band (1450–1700 cm^−1^) [42,43,44,45,46]. The two weak shoulders in the range of 1700–1750 cm^−1^, are attributed to APTES modification [47]. The reducing effect can be recognized by the slightly decreased intensity ratios of D-peak to G-peak (I_D_/I_G_) and the increased 2D peaks [45], as shown in the chart of Figure 2A. However, the relatively larger I_D_/I_G_ also suggests there are many unrecovered defections in rGO-COOH and rGO. Moreover, the full width at half maximum (FWHM) of 2D band is about 170 cm^−1^ for rGO-COOH and GO, as shown by the inset of Figure 2A, which is bigger than the reported FWHM of rGO (78 ± 18 cm^−1^) [45]. This phenomenon is similar to the finding in [45], in which it is attributed to the high temperature induced warping of sp^2^ carbon atoms’ planar network. In this work, this distortion may be related to the low reduction degree, because no further annealing process is executed in this work, then, the disturbed sp^2^ network will hinder the two phonon double resonance process which forms of 2D peak. As a result, the 2D peaks in this work are not sharp.

In Figure 2B, the characteristic UV-vis absorption peaks of the π−π* transition are red shift after reduction and located at ~266.4 nm (rGO-COOH) and ~267 nm (rGO), close to graphene’s feature peak (~270 nm). The reducing effects are evidenced by the red shifts from oxidized state to reduced state (rGO is from ~235 nm to 267 nm, rGO-COOH is from 240 nm to 267 nm), which also demonstrate the damaged conjugated domains in GO-COOH and GO are recovered by the hydrazine reduction [38]. More proofs are provided by the slight variations of shoulders I and II in UV-vis spectra, as shown by the insets in Figure 2B, which are closely associated with oxide defections [38]. Meanwhile, in the shoulder II (~369 nm) only remaining in rGO-COOH also indicates there may be carboxyl related defections, which influence the UV-vis absorption in the region of 368–370 nm, more XPS evidence will be presented in the next section.

Meanwhile, nano-scale flatness can be demonstrated by the AFM image of rGO-COOH film on PET, in contrast with the dotted points in rGO’s AFM photo (as shown in Figure 2C,D). This comparison suggests rGO-COOH is more helpful to form a smoother graphene layer than rGO, which is proofed by the SEM photographs in Figure 2F,G. Besides, similar phenomena are found in glass supported films, as shown in Figure S2C,D, it may be induced by the accumulated oxygen-containing groups at the edge of rGO-COOH flakes [48]. The misty covering layer found in the pGp film’s SEM (Figure S2B), it indicates there is a polymer-like material in pGp which is also confirmed by the following XPS characterizations.

### 3.2. XPS Characterizations

**Figure 3:**
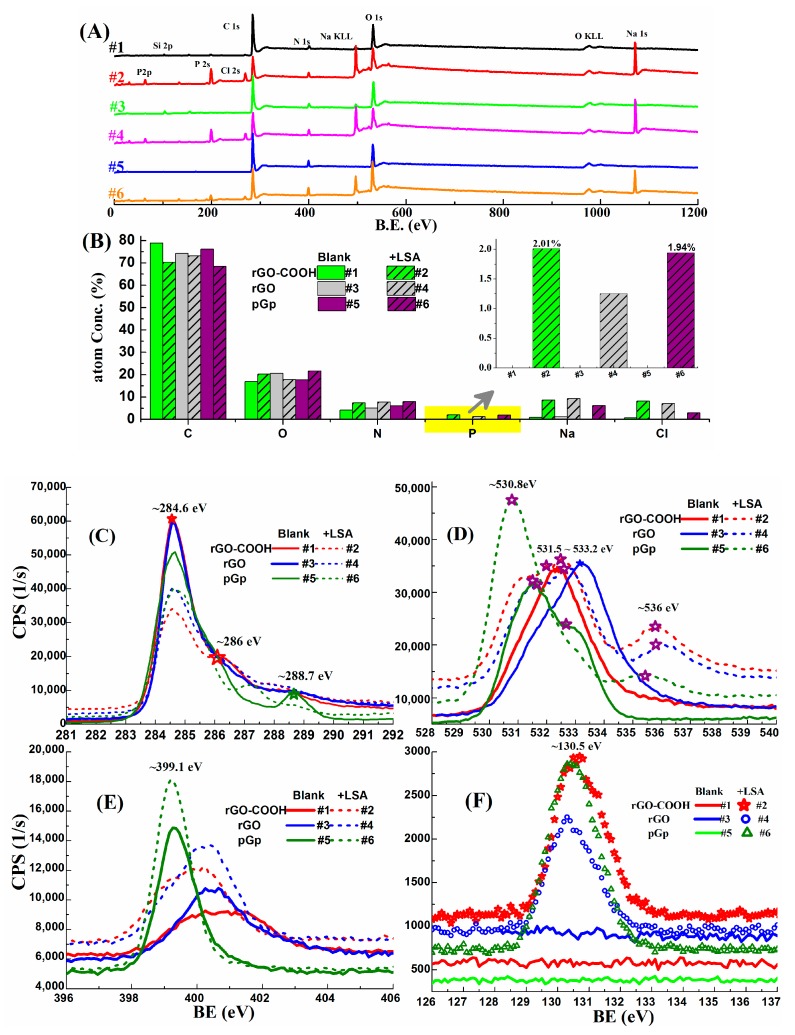
XPS characterizations of rGO-COOH, rGO and pGp film on the APTES modified glass slides before and after LSA immobilization, numbered as #i (i = 1, 2, 3, 4, 5, 6). (**A**) Wide spectra. (**B**) Elemental contents of the samples. (**C**–**F**) C1s, O1s, N1s and P2p core spectra, respectively.

The wide spectra and core spectra (C1s, O1s, N1s and P2p) of the films of rGO-COOH, rGO and pGp (before and after LSA immobilization) are presented in Figure 3; the fitted core spectra are provided in Figures S3–S5, by using quantification tools of Casaxps^®^ 2.3. LSA immobilizations on the samples can be identified by P elemental variations as shown in Figure 3B, because in this experiment the only source of P element is from LSA.

The main elements and their changing traces can be recognized from the wide spectra in Figure 3A. The obvious peaks at around 285, 400 and 535 eV are found in all the six samples, and belong to C1s, N1s and O1s, respectively; they are contributed to graphene, APTES and nucleic acid (LSA). The evolutions of their core spectra reflect the LSA’s coating effects, which are the lowered sp^2^ peaks (~284.6 eV) in C1s core spectra (Figure 3C), the broadened core spectra of O1s and N1s (Figure 3D,E). There is an obvious bump in C1s of pGp (#5), it is assigned to the polyester in pGp, which is used in the paste medium and viewed in its SEM (Figure S2B). Immobilized LSA molecules are evidenced by the split peaks: (1) the C1s components in nucleobase cytosine (C-base, at about 285.0 eV) and bonds of C–N or C–O (at about 286.6 eV) in LSA [49] are identified by the fitted peaks of C1s in Figure S3B,D,F; (2) the extra peaks (@536 eV, in Figure 3D) in #2, #4 and #6 belong to the entrapped water in LSA [50]; (3) the turnout of P2p peaks in #2, #4 and #6, as shown in Figure 3F.

For N1s fitted peaks in Figure S5A,B, peaks at 398.8 eV are attributed to the neutral imine nitrogen groups [51] which may be induced by APTES pretreatment; while the peaks at around 400 eV are related to the C–N bonds, for #1 and #3, they belong to the hydrazine alcohols formed during hydrazine reduction [52]; for #2 and #4, they are mainly contributed to by the N1s components in nucleic acid [49]. While, for N1s core spectra of pGp samples in Figure S5C, the fitted peak at 398.6 eV is assigned to the polymer medium which is found by C1s spectra (Figure 3C) and SEM image in Figure S2B.

The XPS-peak-differentiating analyses of C1s in rGO-COOH and rGO are conducted and presented in Figure S3A,C, the split peak at about 290 eV in #1 demonstrate that there are carboxyl groups in rGO-COOH, in contrast, it is absent from #3 which indicates that the COOH group in rGO is scarce.

### 3.3. Electronic Features

**Figure 4:**
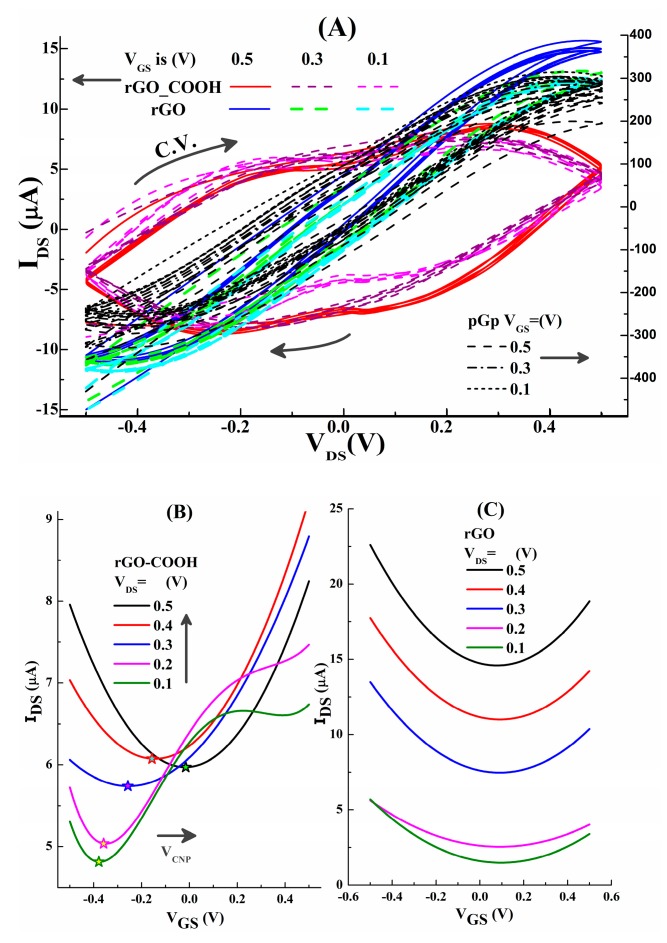
(**A**) Output features of rGO-COOH, rGO and pGp channeled GFETs. (**B**) and (**C**) The transfer curves of rGO-COOH and rGO, respectively.

The basic electronic examinations are executed on unmodified devices (blank) using the measuring setup given in Figure 1C. According to our previous works about GFETs [53], the testing ranges of V_GS_ and V_DS_ are in the range of −0.5–0.5V. The voltage of V_DS_ are swept from −0.5 to +0.5 V, when V_GS_ are maintained at the constant values 0.5, 0.3, 0.1V, respectively, as shown in Figure 4A. At the same time, there are ambipolar transfer characteristic curves in rGO-COOH and rGO channeled devices, as shown in Figure 4B,C. They are fitted from their measured data which are presented in Figure S6, by the use of the “polynomial fit” in Origin^®^. However, for pGp-FET, no ambipolar feature can be measured (as shown by Figure S6C).

Explanations for the measured results are presented according to the classical “site-binding” model [54,55] which has been applied in the analyzation of pH-sensing in rGO-GFET [18]. The equivalent circuit for the device is proposed in Figure S7, the parallel connected resistor (R_EDL_) and capacitor (C_EDL_) are determined by electric-double-layer at the interface of solution and graphene channel; then, there is an extra dropping voltage (V_EDL_) on this layer. Under the same liquid environment, the values of R_EDL_ are constant; the values of C_EDL_ depend on the charges at this interface. The abundance of oxygen containing groups on rGO-COOH (evidenced by XPS in Section 3.2) indicate more charges on it, then, C_EDL_ in rGO-COOH samples would be higher than the others, then more V_EDL_ will be consumed by EDL. Furthermore, I_DS_ can be changed by the varied the effective voltage applied on the channel surface (V_GS,EFF_), V_GS,EFF_ = V_GS_ − V_EDL_ − V_X_, V_X_ is the voltage across the channel. In Figure 4B, the lowest point is corresponding to the charge neutral point (V_CNP_), the positively shifted V_CNP_ points with the increasing of V_DS_ are measured by rGO-COOH devices. This is in agreement with our works about liquid exfoliated graphene (LEG) based solution-gated field effect transistor (SgFET) [53]. However, in Figure 4C, V_CNP_ points of rGO-FET are almost un-shaken and maintained at about 0.1 V. Besides, it is found that the pGp based SgFET has no ambipolar feature. This may be caused by the polymer coating on pGp film, which makes V_CNP_ go beyond our selected range of V_DS_ and V_GS_. For the following examinations, V_GS_ = 0.5 V and V_DS_ = 0.5 V are selected as the bias voltage, which means electrons are the main carriers.

### 3.4. LSA Immobilization and Pb^2+^ Detection

**Figure 5:**
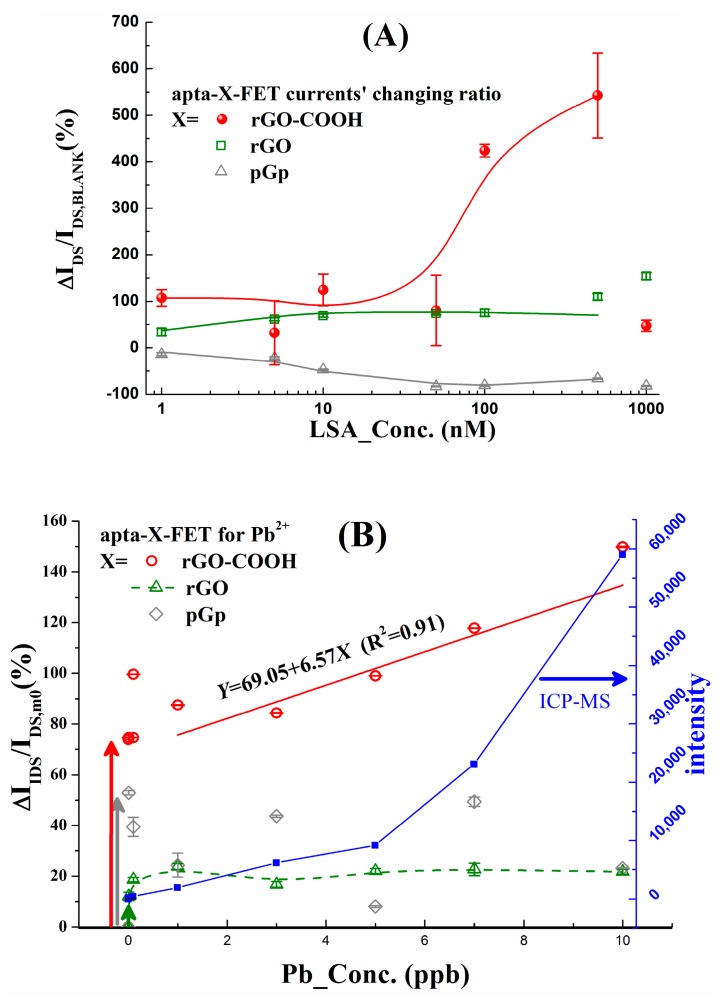
Response for LSA immobilization (**A**) and Pb^2+^ (**B**). Meanwhile, ICP-MS examinations are performed as a control, which are shown by the blue axis in (**B**). In these figures, the values of y-axis in (**A**) and left y-axis in (**B**) have been defined in Section 2.6 and Section 2.7, respectively. The error bar is the relative standard deviation (RSD) and n = 5; R^2^ is the correlation coefficient of the linear fitted line.

The experiments of LSA immobilization and Pb^2+^ detections on the platform of SP-X-FETs (X = rGO-COOH, rGO and pGp) are conducted following the cyclic schemes (illustrated in Section 2.6 and Section 2.7), the results of which are plotted in Figure 5A,B. It is found, when LSA_Conc. is increased from 1 to 500 nmol/L, I_DS_ changing rate (ΔI_DS_/I_DS,BLANK_) of rGO-COOH-FET increases dramatically, in comparison with the data of rGO and pGp; when LSA_Conc. is 1000 nmol/L, it falls close to the data of rGO and pGp.

The response of SP-X-FET for the anchored LSA can be explained in two ways: (1) electron carriers can be injected into the graphene layers through its phosphate backbone, the currents (I_DS_) should be increased by the anchored LSA [56]; (2) in reference with our previous work about functional nucleic acid modified LAPS [36], LSA molecules are in double strand architecture before reaction with Pb^2+^, they will increase the resistor across EDL (R_EDL_). When LSA is in low concentration, the former is dominant, then the currents are increased, as shown by the red curve in Figure 5A; when the LSA concentration is 1000 nmol/L, LSA molecules will be stacked on the first layer LSA, which will increase R_EDL_ and V_EDL_, then the lowered I_DS_ is measured. So, in the following experiments, 500 nmol/L LSA is used to functionalize SP-X-FETs (X = rGO-COOH, rGO, pGp).

For rGO samples (the green square symbols in Figure 5A), there is an up-trend with the increasing of LSA_Conc, when LSA_Conc is 500 nmol/L, the value of ΔI_DS_/I_DS,BLANK_ is close to 100%. That means, there are anchored LSA molecules on rGO through π–π^*^ conjugations, the injected electrons from LSA to rGO channel can increase I_DS_. But the number of LSA molecules on rGO is less than on rGO-COOH one (evidenced by XPS P 2p spectra, Figure 3E), so the increasing of I_DS_/I_DS,BLANK_ for rGO is smaller than rGO-COOH. While, for the data of X = pGp, their variations are reverse to rGO and rGO-COOH, which is caused by the polymer residue on the graphene, evidenced by SEM image (Figure S2B), which hinders the electron injection from LSA molecule to graphene channel.

Subsequently, detections of Pb^2+^ standard samples are executed following the procedure in Figure S1B, the results are presented in Figure 5B, and compared with the conventional ICP-MS method shown by the right y-axis. It can be identified from the data of apta-X-FET (X = rGO-COOH, rGO and pGp), the highest responding for Pb^2+^ and the best coincidence with ICP-MES results are displayed by X = rGO-COOH (the red circle symbols) with the limit-of-detection (LOD) of 0.001ppb, the linear fitted line is obtained in the range of 1–10 ppb with the sensitivity of 6.57 %/ppb (R^2^ = 0.91). The sensing principle of the apta-X-FETs for Pb^2+^ is outlined at here based on the illustrated mechanism in Section 3.4, as well as our previous work about Pb^2+^ mediated cleavage effect on LAPS [36]. LSA’s sensing action can change I_DS_ by two ways: (1) after reaction with Pb^2+^, LSA molecule’s substrate chain (mentioned in Section 2.1) is left, it is a flexible single string and tends to lie on the graphene channel, then more electrons are implanted into the channel; (2) the LSA induced R_EDL_ (in Figure S7) after being cut by Pb^2+^ becomes negligible, because the remaining LSA part is so tender that it cannot stand on the channel anymore; then, V_EDL_ will be reduced. Both of them tend to increase I_DS_, so the increased ΔI_DS_/I_DS,m0_ is measured during Pb^2+^ detections.

In Figure 5B, for apta-pGp-FETs, because of the polymer residue on the channel film, no sensitivity can be obtained; for rGO, there is an increasing trend from 0 to 1 ppb (green arrow in Figure 5B), but the amplitude is dramatically lower than rGO-COOH, that is just in agreement with the few LSA molecule anchored on it, which is evidenced by P2p core spectra, in Figure 3E. In stark contrast, ΔI_DS_/I_DS,m0_ of rGO-COOH based apta-FETs are more than doubly increased, even when Pb_Conc. is 0.001 ppb there is an increase of 73.8%, as shown by the red arrow in Figure 5B.

### 3.5. Selectivity and Real Application

**Figure 6:**
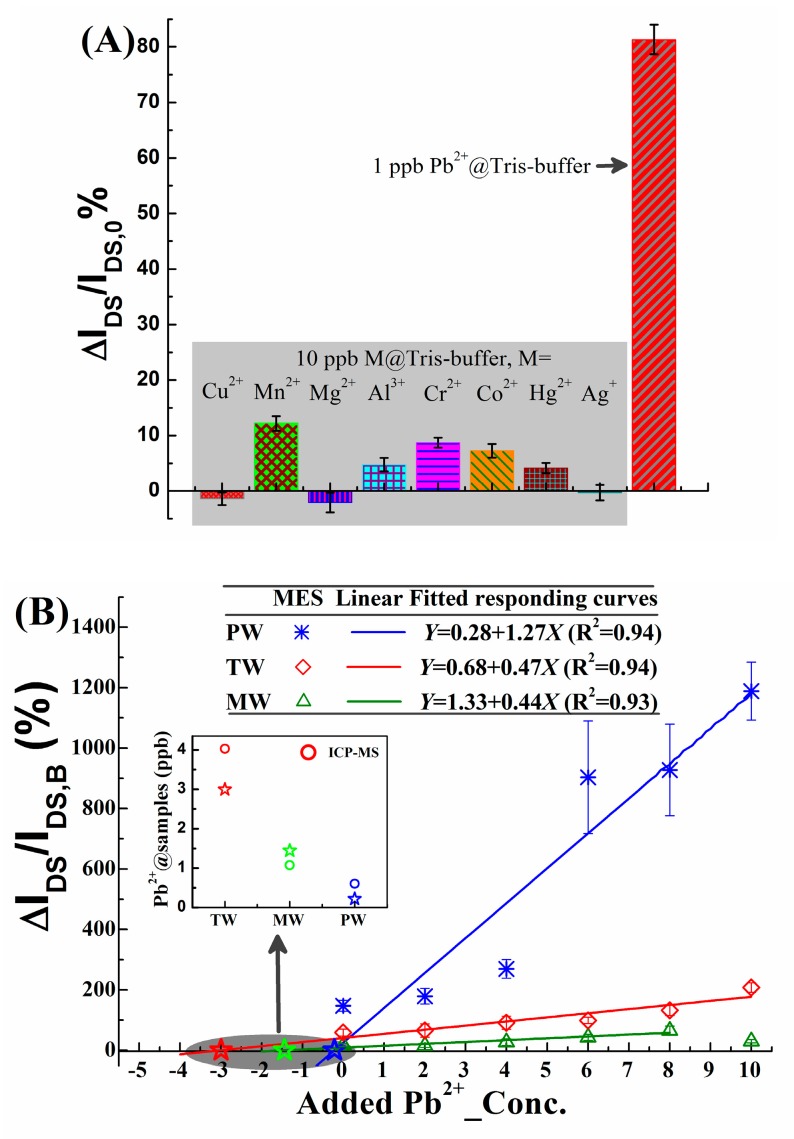
Application tests for the proposed rGO-COOH based GFET as an apta-sensor. (**A**) The specificity for Pb^2+^ is verified by the examinations for un-target metal ions (M = Cu^2+^, Mn^2+^, Mg^2+^, Al^3+^, Cr^2+^, Co^2+^, Hg^2+^, Ag^+^), respectively. (**B**) The measured I_DS_ response for the added Pb^2+^ standard samples in the tap water (TW) from municipal supply, as well as marketed mineral water (MW) and pure water (PW). Error bar is RSD and n = 5. The inset in (B) is the calculated Pb^2+^ concentrations in three samples (Pb^2+^@samples), according to SAM (details are in Section 2.7); while the ICP-MS measured results for the same samples are plotted in the same coordinate to make a comparison.

The selectivity of the proposed apta-rGO-COOH-FET for Pb^2+^ is examined when disturbing metal ions (M = Cu^2+^, Mn^2+^, Mg^2+^, Al^3+^, Cr^2+^, Co^2+^, Hg^2+^, Ag^+^) are concentrated at 10 ppb and Pb^2+^ concentration is 1 ppb, as shown in Figure 6A. An acceptable specificity is proofed, because the I_DS_ changing rates for Ms are less than one eighth of ΔI_DS_/I_DS,0_ for Pb^2+^, when their concentrations are decuple that of Pb^2+^.

The real applications are executed following the method mentioned in Section 2.7, to confirm the practicability of this self-activated rGO-COOH to serve as a channel material in an Sg-FET apta-sensor. As shown in Figure 6B, sensitivities for the added Pb^2+^ are lowered when the backgrounds of Pb^2+^ are changed from tris-buffer to PW, TW and MW. That suggests the sensing activity of LSA to Pb^2+^ is lowered, because of the complicated ionic composition of these samples. Nonetheless, there are still acceptable linearities as shown by the fitted lines in Figure 6B, which are 1.27, 0.47 and 0.44 %/ppb (R^2^ > 0.9) for PW, TW and MW, respectively. Finally, the calculated concentrations of Pb^2+^ in the real samples are obtained following the method given in Section 2.7. The data from the conventional ICP-MS method (as shown in the inset of Figure 6B) are used as control. The agreement between the two approaches demonstrates that the application of rGO-COOH as a self-activated channel film in the screen-printable apta-FET is a good idea. Meanwhile, the total liquid-chemical procedures in preparing rGO-COOH channeled SP-FET make it compatible with the rapidly developed printing electronics field.

## 4. Conclusions

In conclusion, the studies about exploiting and evaluating rGO-COOH as a self-activated channel material in SP-apta-FET are presented in this work, including methods for material synthesis and device preparation, examinations and analyses for identifying graphene-state, carboxyl group and its augment for LSA immobilization; as well as the electric experiments to demonstrate its feasibility and high efficiency as a self-activated channel material in SP-apta-FET. It is evidenced that the LSA molecules can be grafted onto the rGO-COOH film without any extra activating reagent. The comparisons of LSA functionalized SP-X-FETs (X = rGO-COOH, rGO and pGp) indicate, for the application of aptamer sensing, there should be as many naked surface activating groups as possible on the channel surface, the proposed rGO-COOH is an optimum nominee because of its plenty-of carboxyl groups and free-of polymer residue. Furthermore, theoretical deductions are provided for the measured rGO-COOH enhanced current response for aptamer functionalization and target ion’s sensing. All these works demonstrated rGO-COOH is a promising self-activated channel material for SP-apta-FET, on this platform the amino-modified aptamer probes can be directly anchored on it independent of additional linking process and free-of polymer residue. Benefited by the naked activating sites on rGO-COOH based SP-FET, the charge transfer between the LSA molecules and rGO-COOH channel can be formed, then good sensing performances are achieved in the experiments of both the standard samples and the real samples. According to this work, it is believed rGO-COOH, as a simple graphene derivative, will play an important role in SP-FETs and push the development of their large-scale production and commercialization.

## Supplementary Materials

The following are available online at https://www.mdpi.com/2072-666X/10/6/388/s1, Figure S1: Cyclic experiments for LSA immobilization and the detection of standard Pb2+ solutions, Figure S2: Additional characterizations of Raman and SEM, Figure S3: The C1s core spectra and their peak-fitting curves, Figure S4: Fit analysis of O1s core spectra for XPS sampls, Figure S5: Fit analysis for N1score spectra for the six samples, Figure S6: Electronic transferring measurements of the studied rGO-COOH based SgFET and its counterparts based on rGO and purchased graphene paste (pGp), Figure S7: Diagram of principle in SgFET. Supplementary data related to this article can be found at online Library of Carbon.
