# Supplementary material for: Reduced Carboxylate Graphene Oxide based Field Effect Transistor as Pb2+ Aptamer Sensor
Source: Micromachines (Basel). 2019 Jun 11;10(6):388. doi: 10.3390/mi10060388 (PMC6630652; doi:10.3390/mi10060388)
Supplement: Supplementary file 1 [file micromachines-10-00388-s001.pdf]

Supplementary Materials: Reduced Carboxylate Graphene Oxide based Field Effect Transistor as Pb^{2+} Aptamer Sensor

Fang Li, Zhongrong Wang and Yunfang Jia

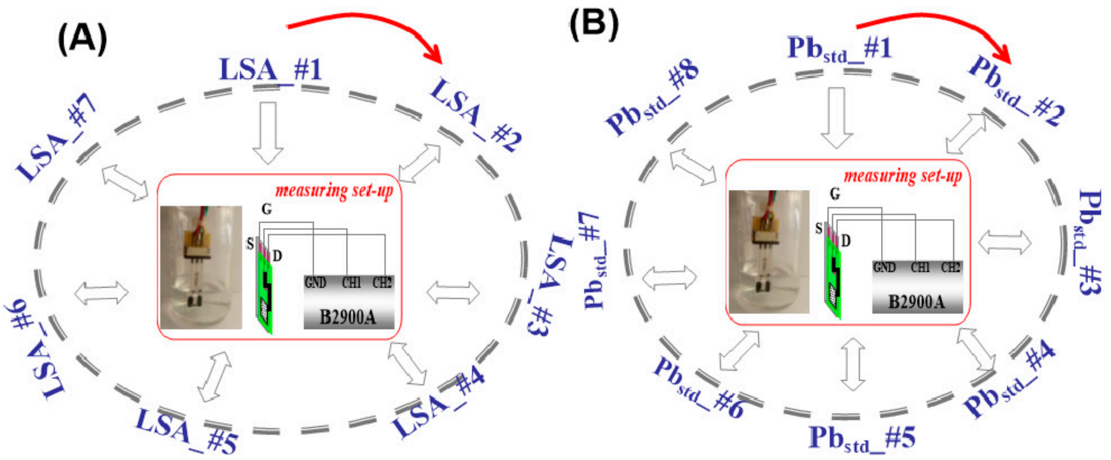

Figure S1. Cyclic experiments for LSA immobilization (A) and the detection of standard Pb^{2+} solutions (B). It needs to note, in these experiments, the electric measuring operations are separated from the incubation steps; furthermore, after being incubated and rinsed, the currents (I_{DS}) of the devices are detected under a constant biochemical environment (similar buffer solution, same volume of solution, similar room temperature, etc.) and electronic bias conditions including (V_{DS} and V_{GS}). The purpose of these arrangements is to make the best possible to ensure the measured variations of I_{DS} are induced by our concerned elements, which are LSA immobilization (A) and lead ions mediated cleavage of LSA (B), respectively.

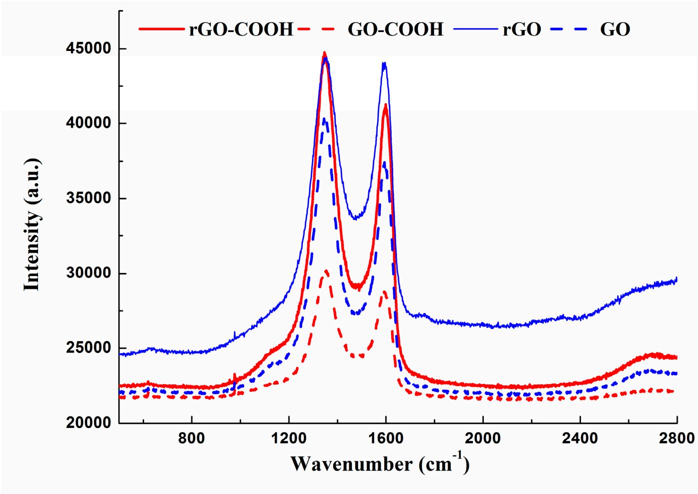

(A)

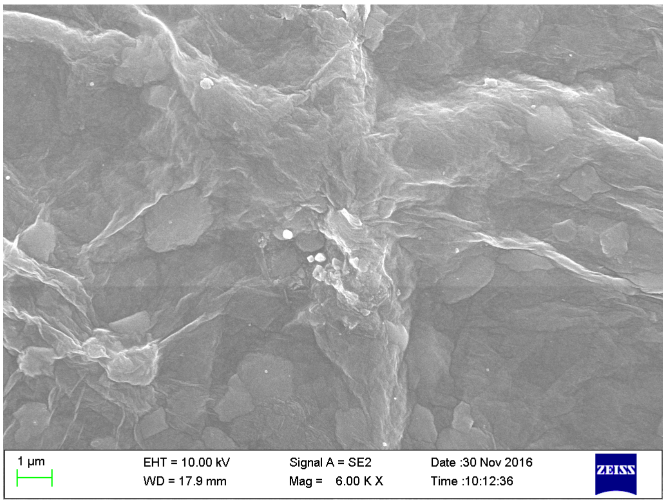

(B)

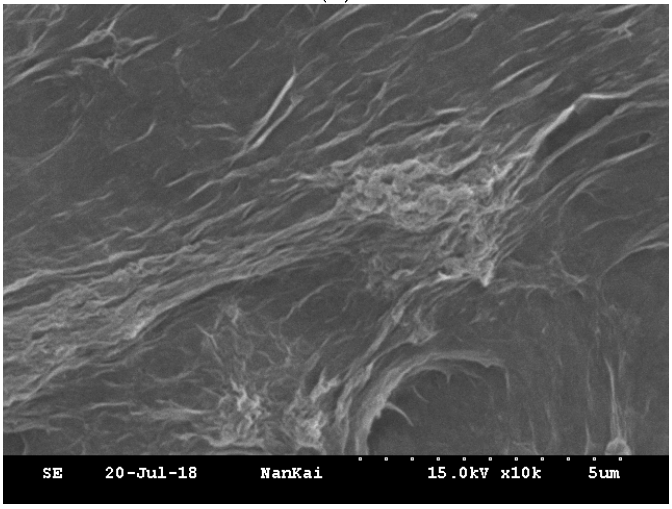

(C)

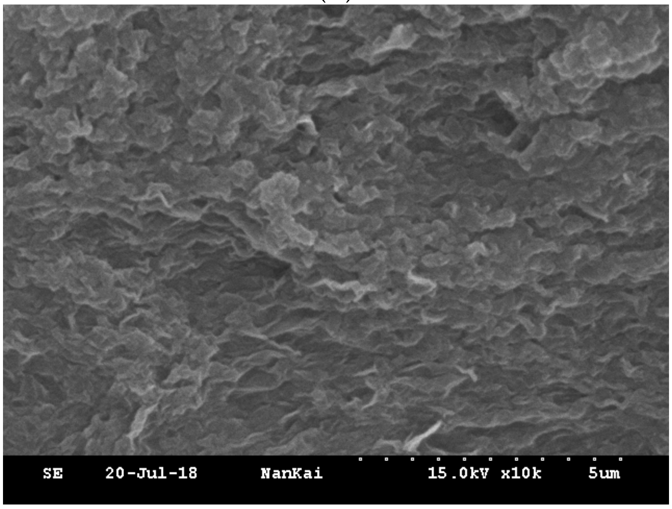

(D)

Figure S2. Additional characterizations of Raman and SEM. (A) Original Raman spectra of rGO-COOH, GO-COOH, rGO and GO, excited at 532 nm. (B) to (D) are SEM images of purchased graphene paste, rGO-COOH and rGO on APTES modified glass substrate, respectively.

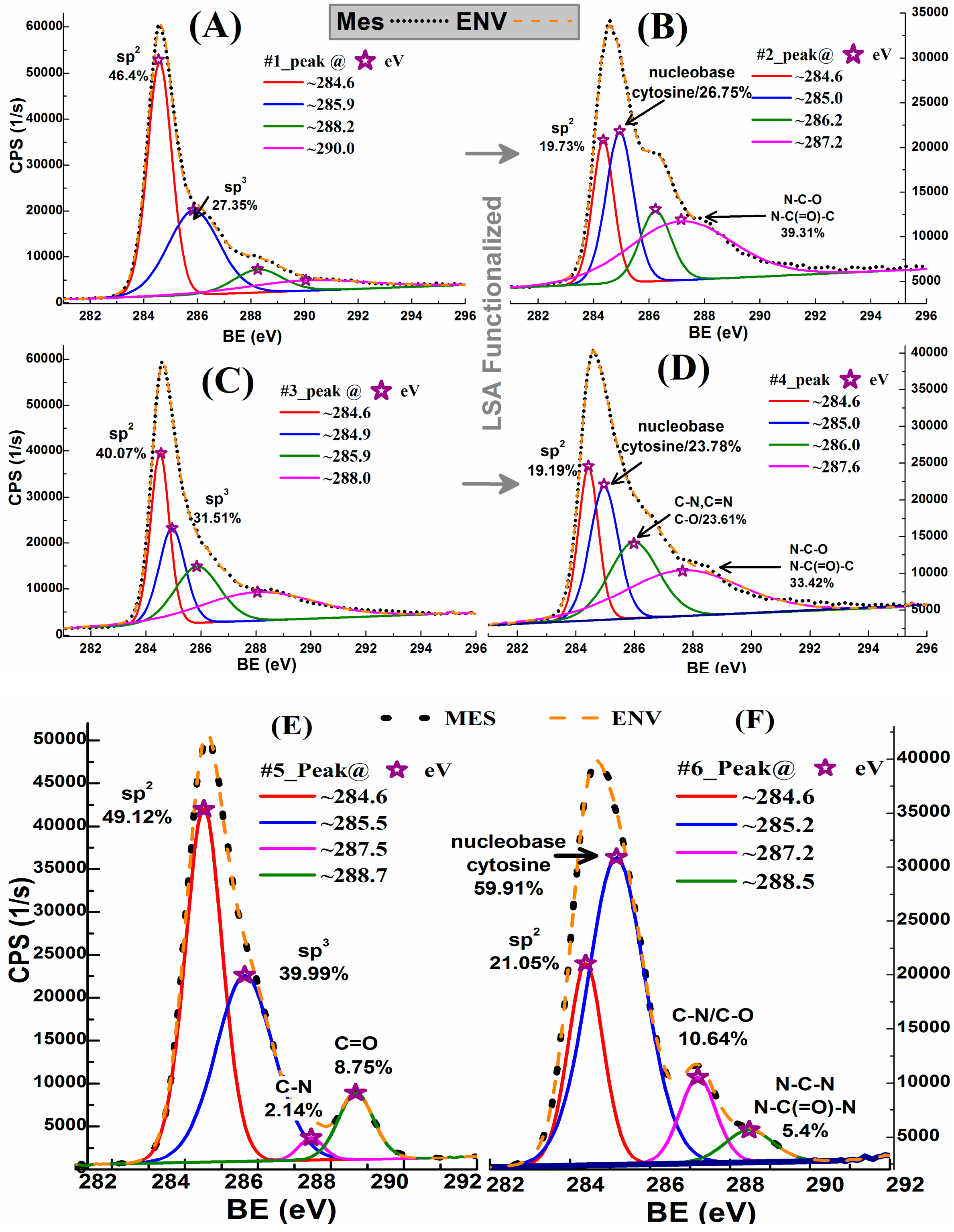

Figure S3. The C1s core spectra and their peak-fitting curves of #1 to #6 in (**A**) to (**F**).

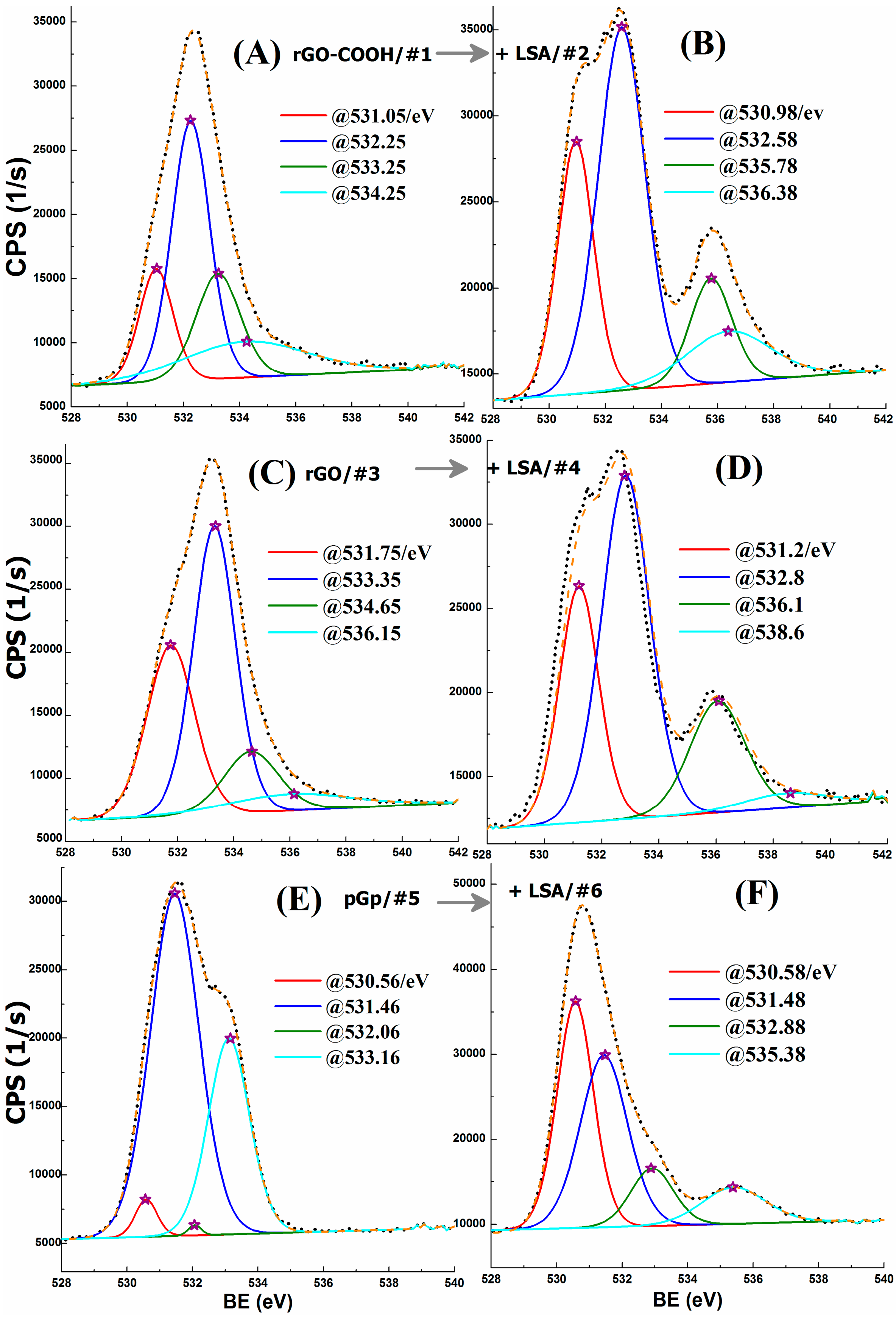

Figure S4. Fit analysis of O1s core spectra for XPS sampls #1 to #6: (**A**) to (**F**).

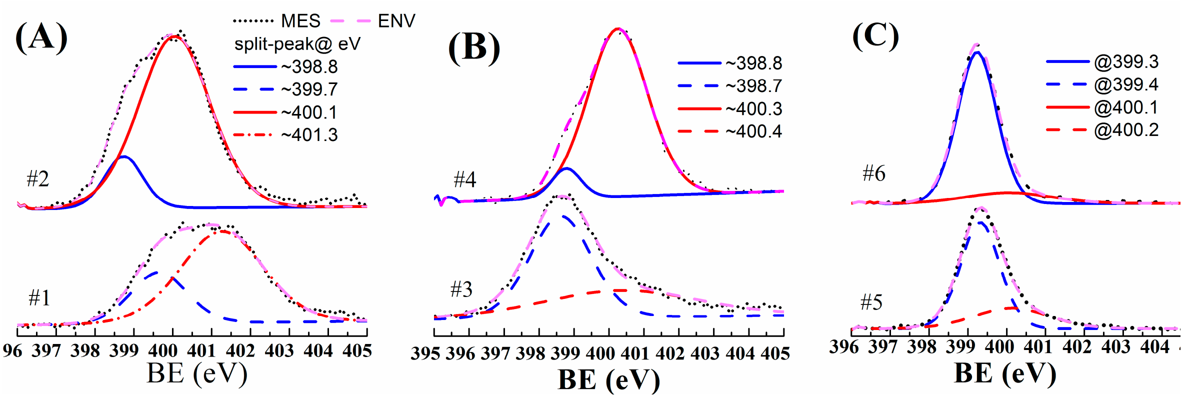

Figure S5. Fit analysis for N1score spectra for the six samples, being grouped as (**A**) for #1 and #2, (**B**) for #3 and #4, (**C**) for (5) and (6).

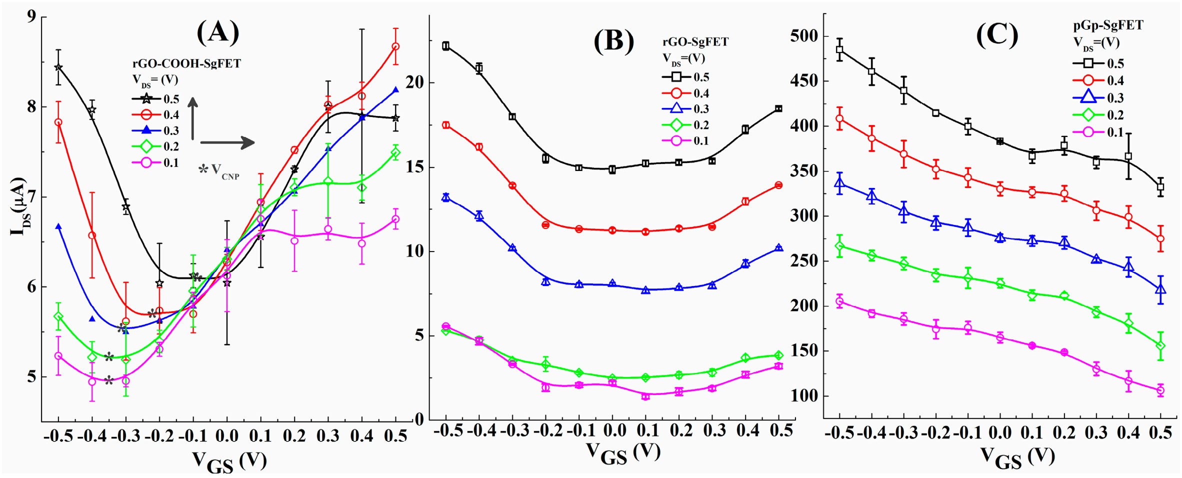

Figure S6. Electronic transferring measurements of the studied rGO-COOH based SgFET (**A**) and its counterparts based on rGO (**B**) and purchased graphene paste (pGp) (**C**). Error bar is the relative standard deviation (RSD) with n = 5.

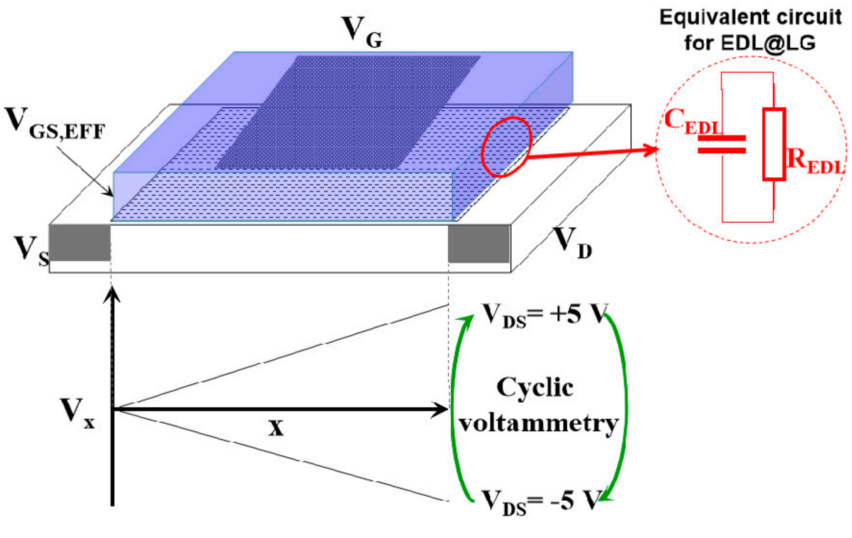

Figure S7. Diagram of principle in SgFET.
